# Wip1 and p53 contribute to HTLV-1 Tax-induced tumorigenesis

**DOI:** 10.1186/1742-4690-9-114

**Published:** 2012-12-21

**Authors:** Linda Zane, Junichiro Yasunaga, Yu Mitagami, Venkat Yedavalli, Sai-Wen Tang, Chia-Yen Chen, Lee Ratner, Xiongbin Lu, Kuan-Teh Jeang

**Affiliations:** 1 Molecular Virology Section, Laboratory of Molecular Microbiology, the National Institutes of Allergy and Infectious Diseases, the National Institutes of Health, Bethesda, Maryland, 20892-0460, USA; 2 Laboratory of Virus Control, Institute for Virus Research, Kyoto University, Kyoto, Japan; 3 Department of Medicine, Washington University School of Medicine, Saint-Louis, Missouri, USA; 4 Department of Cancer Biology, the University of Texas MD Anderson Cancer Center, Houston, Texas, USA

## Abstract

**Background:**

Human T-cell Leukemia Virus type 1 (HTLV-1) infects 20 million individuals world-wide and causes Adult T-cell Leukemia/Lymphoma (ATLL), a highly aggressive T-cell cancer. ATLL is refractory to treatment with conventional chemotherapy and fewer than 10% of afflicted individuals survive more than 5 years after diagnosis. HTLV-1 encodes a viral oncoprotein, Tax, that functions in transforming virus-infected T-cells into leukemic cells. All ATLL cases are believed to have reduced p53 activity although only a minority of ATLLs have genetic mutations in their p53 gene. It has been suggested that p53 function is inactivated by the Tax protein.

**Results:**

Using genetically altered mice, we report here that Tax expression does not achieve a functional equivalence of p53 inactivation as that seen with genetic mutation of p53 (i.e. a *p53*^*−/−*^ genotype). Thus, we find statistically significant differences in tumorigenesis between *Tax*^*+*^*p53*^*+/+*^*versus Tax*^*+*^*p53*^*−/−*^ mice. We also find a role contributed by the cellular Wip1 phosphatase protein in tumor formation in Tax transgenic mice. Notably, *Tax*^*+*^*Wip*1^*−/−*^ mice show statistically significant reduced prevalence of tumorigenesis compared to *Tax*^*+*^*Wip*1^*+/+*^ counterparts.

**Conclusions:**

Our findings provide new insights into contributions by p53 and Wip1 in the *in vivo* oncogenesis of Tax-induced tumors in mice.

## Background

Human T-cell Leukemia Virus type 1 (HTLV-1) is the first identified human retrovirus. The virus belongs to the deltaretrovirus family and is the etiological agent of a highly aggressive neoplastic disease, Adult T-cell Leukemia/Lymphoma (ATLL), and inflammatory diseases including HTLV-1 Associated Myelopathy (HAM)/Tropical Spastic Paraparesis (TSP), uveitis, infective dermatitis and myositis [1-9]. HTLV-1 infects approximately 20 million individuals world-wide, and 1-5% of infected individuals will develop ATLL after a long latency period of 20 to 60 years [1].

HTLV-1 encodes a viral Tax oncoprotein. The singular expression of Tax is sufficient to transform primary rodent cells [10] and potentially human embryonic stem cells [11], immortalize human primary T lymphocytes [12,13], and induce tumors in transgenic mice [14-17]. Tax confers pro-proliferative and pro-survival properties to HTLV-1 infected cells [18-20] by pleiotropically activating effector proteins including the Cyclic AMP Responsive Binding Protein (CREB) and CBP/p300 [21-24], Nuclear Factor kappa-B (NF-κB) [25-29], Cyclin-Dependant Kinases (CDKs) [30-33], and Akt [34-36] amongst others. Tax also triggers DNA damage [37-42]. In transforming a normal T-cell into a leukemic cell, it is believed that Tax must also neutralize cellular checkpoints (e.g. p53 and mitotic spindle assembly checkpoint) that act to censor DNA damage [43,44] and aneuploidy [45,46].

p53 is a DNA-binding transcription factor that plays a key role in cell cycle regulation, apoptosis, and DNA repair [47]. The p53 gene is recognized as one of the most important tumor suppressor genes and is frequently mutated in human tumors including hematologic malignancies [48-50]. In many human malignancies, the frequency of p53 genetic mutation is ≥50% [51,52]; however, the frequency of mutated p53 in ATL patients is reported to be around 15% [53-58], suggesting that loss of p53 activity in ATL may largely arise through a mechanism other than genetic mutation. Several *in vitro* studies in different cell types have shown that Tax represses p53 activity [59-65]. Various mechanisms have been proposed for Tax-inactivation of p53. Indeed, it has been suggested that Tax inactivates p53 by acting through either the CREB [62] or the NF-κB [66,67] pathway; however, it has also been noted that neither mechanism satisfactorily explains Tax-p53 interaction [65], leaving the question of how Tax effectively disables p53 function incompletely answered.

Here, we have conducted *in vivo* experiments in mice to address two questions. First, we have assessed the effectiveness of Tax mediated inactivation of p53 *versus* inactivation of p53 by genetic mutations. Second, we have characterized Wip1 as a cooperating *in vivo* Tax co-factor in p53 inactivation. Using various genetically altered mice, we show that Tax inactivation of p53 is functionally less stringent than p53 inactivation by genetic mutation, and we report that the cellular Wip1 phosphatase protein collaborates functionally with Tax in inhibiting p53 activity.

## Results

### *Tax*^*+*^*p53*^*–/–*^ mice show reduced tumor free survival compared to *Tax*^*+*^*p53*^*+/+*^

**Figure 1:**
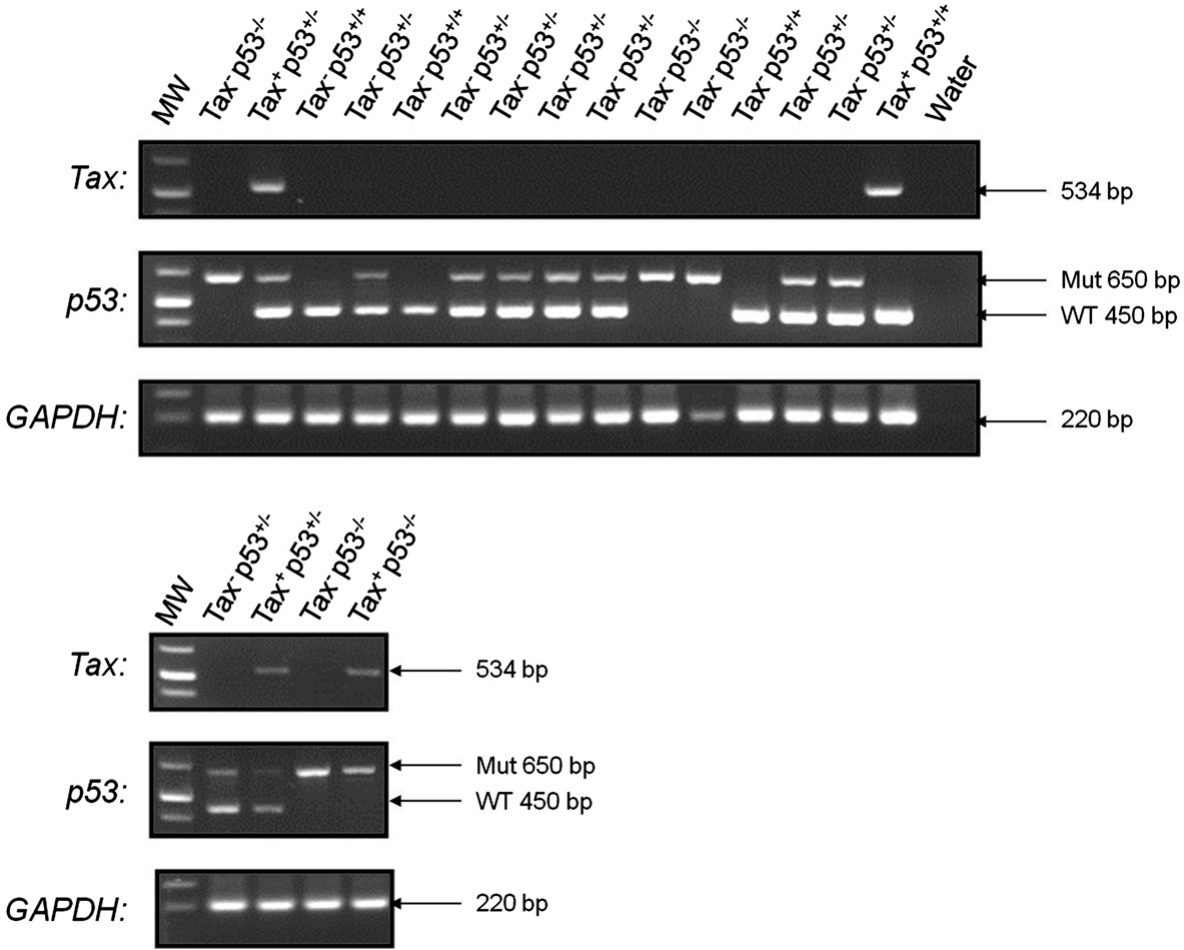
**Genotyping of *p53KO/Tax Tg *mice.***p53* primers distinguish between WT and mutant p53 alleles with PCR products of 450 and 650 bp in size (top), respectively. Middle panel shows the detection of Tax DNA (534 bp), and the bottom panel shows PCR control detecting cell endogenous GAPDH gene (220 bp). Mut, mutant ; WT, wild type. Tax, p53, and GAPDH signals are as indicated.

**Figure 2:**
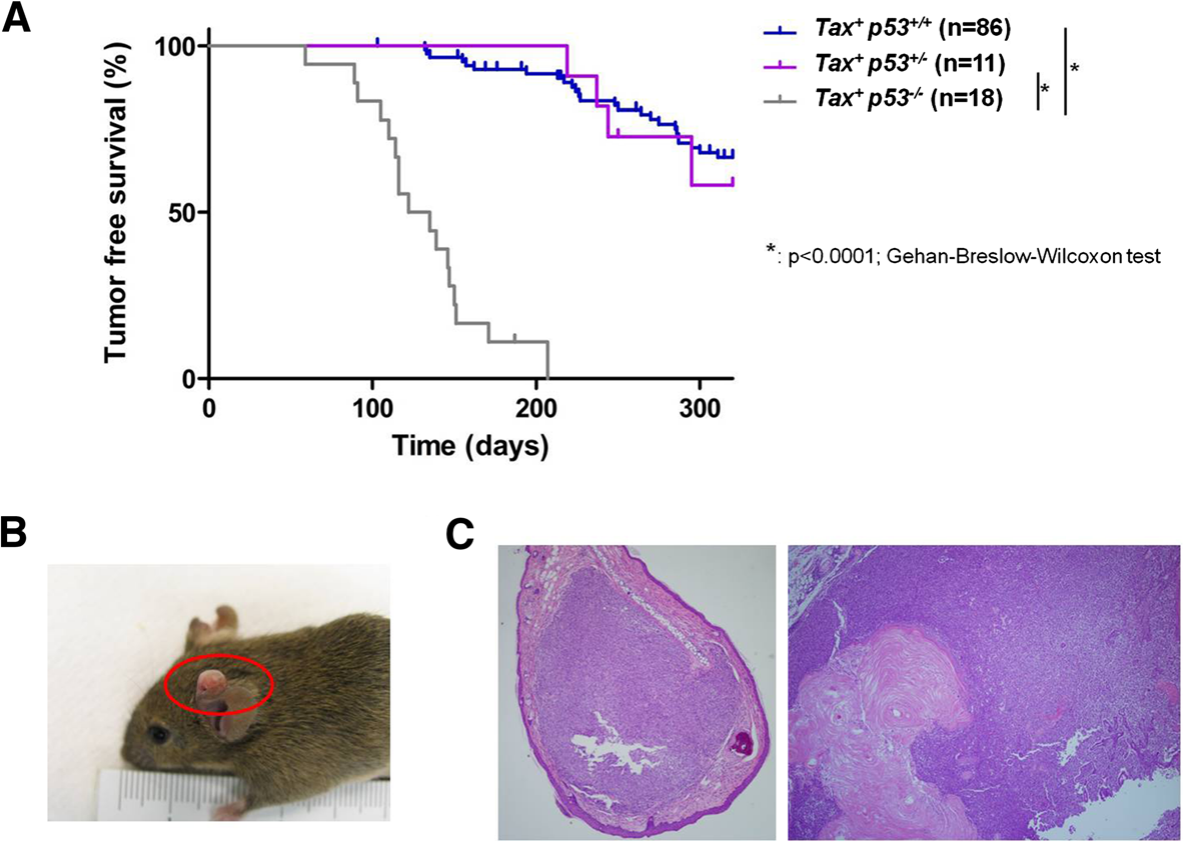
**Statistically significant reduction of tumor-free survival in *Tax***^***+***^***p53***^***−/−***^**mice compared to *Tax***^***+***^***p53***^***+/+***^**.** (**A**) Tumor-free survival curves show decreased tumor free survival in *Tax*^*+*^*p53*^*−/−*^ mice compared to *Tax*^*+*^*p53*^*+/−*^ or *Tax*^*+*^*p53*^*+/+*^ animals. Statistical significance (*: p<0.0001) between Tax^*+*^*p53*^*−/−*^ and either *Tax*^*+*^*p53*^*+/−*^ or *Tax*^*+*^*p53*^*+/+*^ mice was determined using Gehan-Breslow-Wilcoxon test. (**B**) A Tax transgenic mouse with an ear tumor is shown for illustration. (**C**) Examples of tumor histology [hematoxylin and eosin (H&E) staining] from *Tax*^*+*^*p53*^*+/+*^ mice are shown. Example of a pleomorphic ear sarcoma from a *Tax*^*+*^*p53*^*+/+*^ mouse; (left): expansive soft tissue tumor located in peripheral connective tissue of the ear beneath the supportive cartilage; example of a hind leg pleomorphic histiocytic sarcoma and glandular adenocarcinoma with squamous hyperkeratosis from a *Tax*^*+*^*p53*^*+/+*^ mouse (right): the sarcoma consisted primarily of histiocytic tumor cells with dispersed round cells, sparse spindle cells and neutrophilic granulocytes. Similar tumors are also seen in *Tax*^*+*^*p53*^*−/−*^ mice.

In ATLs, p53 genetic mutations are less frequent than those seen in many other cancers [53,54,58]. It has been reasoned that the ability of Tax to inacti-vate p53 function [55] explains why ATL cells may not need to inactivate p53 by genetic mutation. Nevertheless, it has not been clearly characterized whether Tax inactivation of p53 is quantitatively equivalent to inactivation of p53 by genetic mutation. We sought to investigate this issue using gene-tically altered mice. Accordingly, we crossed Tax transgenic mice [15] with *p53*^*−/−*^ mutant mice [68] to generate *Tax*^*+*^*p53*^*−/−*^, *Tax*^*+*^*p53*^*+/−*^ and *Tax*^*+*^*p53*^*+/+*^ progenies. We analyzed the genotypes (Figure 1) of the offsprings and monitored the animals over >300 days for tumor development (Figure 2). Tumor-free survival for *Tax*^*+*^*p53*^*−/−*^ mice (Figure 2A) was significantly worse compared to *Tax*^*+*^*p53*^*+/−*^ and *Tax*^*+*^*p53*^*+/+*^ counterparts (p < 0.0001; Gehan-Breslow-Wilcoxon test). There were no statistically significant differences in the levels of Tax expression between these two categories of Tax^+^ mice supporting that the difference in tumor-free survival was not due to levels of Tax expression (Additional file 1: Figure S1). Interestingly, no significant difference in tumor-free survival between *Tax*^*+*^*p53*^*+/−*^ and *Tax*^*+*^*p53*^*+/+*^ mice was found (p = 0.7093; Gehan-Breslow-Wilcoxon test); this finding agrees with our previous tumorigenesis study of *p53*^*+/−*^ and *p53*^*+/+*^ mice [69] that, in the context of our mice, we find no significant functional difference between homozygosity *versus* heterozygosity in wild type p53. Thus, our finding of a distinct difference in tumor-free survival of *Tax*^*+*^*p53*^*−/−*^ compared to *Tax*^***+***^*p53*^*+/+*^ mice indicates that Tax inactivation of p53 (i.e. *Tax*^***+***^*p53*^*+/+*^) is qualitatively less stringent than genetic inactivation of p53 (i.e. *Tax*^***+***^*p53*^*−/−*^).

### Wip1 phosphatase modulates p53 activity

**Figure 3:**
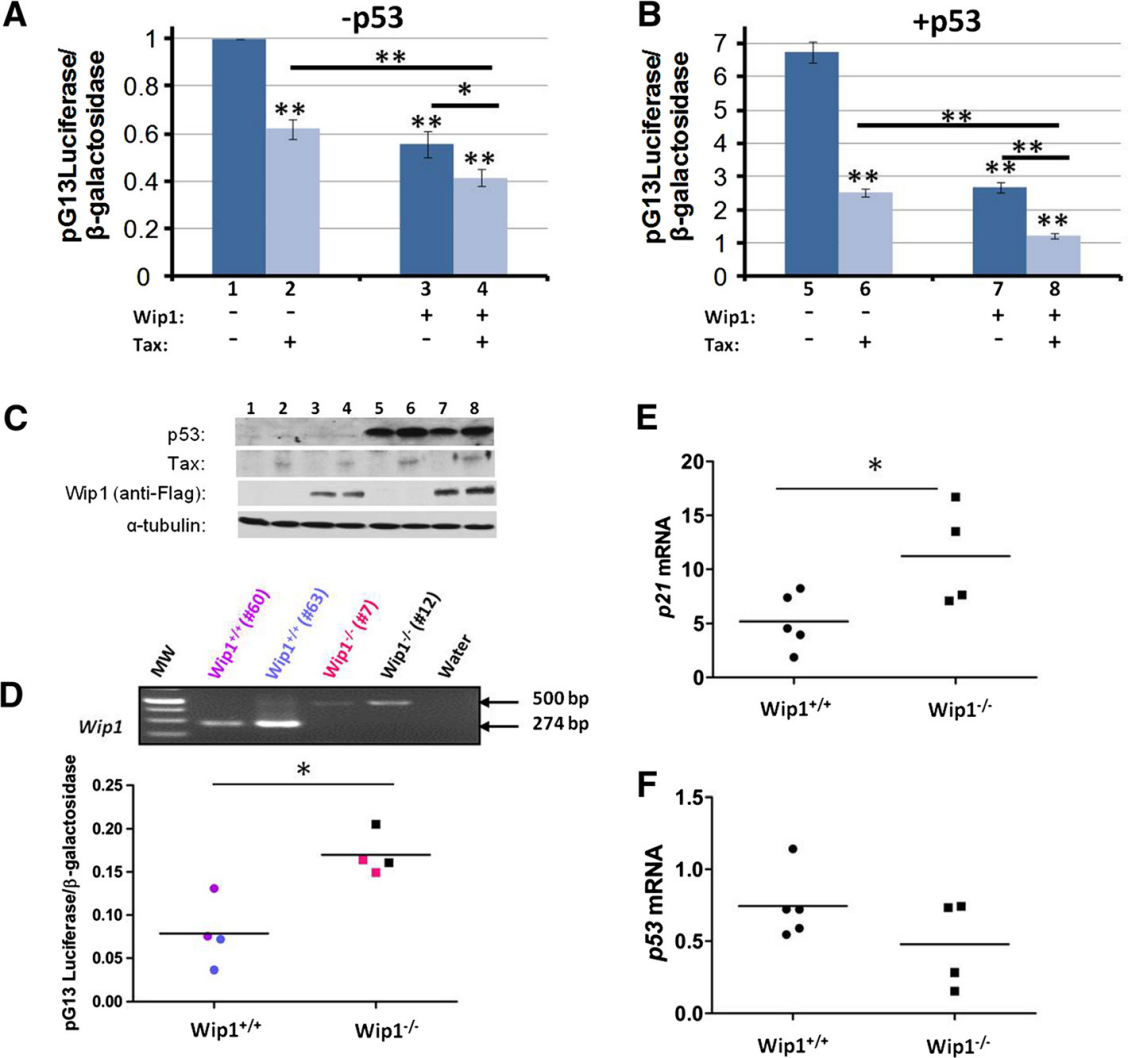
**Wip1 phosphatase attenuates p53 activity.** Wip1 and/or Tax expression reduces p53 transactivation of a pG13Luc-reporter in HCT-116 cells in the absence (**A**) or in the presence (**B**) of exogenous p53 (0.8 μg). HCT-116 cells were transfected with 0.2 μg of Tax and/or 0.75 μg of Wip1 expression plasmid (*: 0.01≤p≤0.05; **: p<0.05; t-test). (**C**) Cell lysates from a representative experiment were subjected to immunoblotting using anti-p53, anti-Tax, anti-Flag and anti-α-tubulin as indicated. The lane numbers of the samples in each case corresponds to the lane numbers indicated in panels (**A**) and (**B**). (**D**) Analysis of cell endogenous p53 activity was conducted using the pG13-Luciferase plasmid in *Wip1*^*−/−*^ and *Wip1*^*+/+*^ Mouse Embryonic Fibroblasts (MEF). Top panel shows PCR genotypic characterizations of two independent Wip1^+/+^ (60, 63) and two independent Wip1^−/−^ (7, 12) MEFs; each was assayed twice in pG13Luc-reporter assays. Bottom graph shows the luciferase assays. All luciferase activities were normalized to a co-transfected β-galactosidase reporter. Statistical significance was determined using t-test (*: p=0.0076). (**E**) Analyses of cell endogenous p21 and (**F**) p53 mRNAs in 5 independent Wip1^+/+^ (left) and 4 independent Wip1^−/−^ MEFs. Real-time RT-PCR analyses of *p53* and *p21* and *GAPDH* (internal standard) transcripts were performed in *Wip1*^*−/−*^ and *Wip1*^*+/+*^ MEFs. There was no statistically significant difference in p53 mRNA levels, while p21 mRNA levels were significantly different between *Wip1*^*+/+*^ and *Wip1*^*−/−*^ MEFs (*: p=0.0425; t-test).

We wished next to understand how other non-genetic means of inactivating p53 might cooperate with Tax in cellular transformation. Wip1 (Wild-type p53-induced phosphatase 1) is a human protein phosphatase that has been shown to be amplified and over-expressed in multiple human cancers and has been suggested to exhibit oncogenic potential [70]. A plausible mechanistic scenario could be that Wip1 acts to inhibit p53 activity, thereby contributing to tumorigenesis. Through its ability to inhibit p53 tumor suppressor function, Wip1, like Tax, may reduce the selective pressure for *p53*-inactivating mutations during cancer progression [71,72]. To check the effect of Wip1 on p53, we assessed how its over-expression affects p53’s transcriptional activity. Accordingly, we transfected human HCT-116 cells with a luciferase reporter plasmid containing 13 copies of a p53 consensus binding site (pG13-Luc; [73]) together with a Wip1 expression plasmid (Figure 3A and B), or we transfected pG13-Luc with a Tax expression plasmid-alone, or we transfected pG13-Luc with both Wip1 and Tax expression plasmids (Figure 3A and B). Under our transfection conditions, both Wip1-alone and Tax-alone with pG13-Luc robustly repressed the expression of the reporter plasmid by more than 40% (p=1.496 × 10^-5^ for Wip1-alone; p=7.62×10^-5^ for Tax-alone; t-test) (Figure 3A). Of note, the co-transfection of Wip1 with Tax repressed pG13-Luc expression by an additional 20% and 15% over that achieved with Tax-alone (p=0.0025; t-test) or Wip1 alone (p=0.019; t-test) (Figure 3A). When the transfections were performed in the presence of co-introduced exogenous p53, we again observed a statistically significant repression of p53 transcriptional activity; here, we saw >60% repre-ssion of pG13-Luc expression after transfection with Wip1-alone (p=3.27 ×10^-5^; t-test) or Tax-alone (p=2.22 ×10^-5^; t-test) (Figure 3B). In the presence of exogenously introduced p53, the co-transfection of Wip1 and Tax repressed pG13-Luc expression by more than 50% over that achieved with Tax-alone (p=7.43×10^-5^; t-test) or Wip1-alone (p=1.25×10^-4^ t-test) (Figure 3B). In Figure 3C, the expression of the transfected plasmids used in Figures 3A and 3B was checked by Western blotting. Taken together, these findings support that Wip1 and Tax cooperate in overall p53 inactivation.

Transient over-expression assays generally are imperfect reflections of physiological regulation. To ask in a more physiological manner how endogenous Wip1 expression regulates p53 activity, we independently isolated several primary MEF clones from *Wip1*^*−/−*^ knock-out mice [74] and their *Wip1*^*+/+*^ wild type siblings (genotyping examples of MEFs are shown in Figure 3D, top). We then compared cell endogenous p53 activity in several independently isolated *Wip1*^*−/−*^ MEFs to other independently isolated control *Wip1*^*+/+*^ MEFs employing either the pG13-Luc reporter assay (Figure 3D, bottom) or by determining the mRNA expression levels of a known p53-responsive target gene, p21^WAF1/CIP1^ (Figure 3E). Notably, the *Wip1*^*−/−*^ MEFs showed statistically significant higher levels of pG13-Luc expression (p=0.0076; t-test) and higher levels of p21 mRNA (p=0.0425; t-test) than the *Wip1*^*+/+*^ MEFs, suggesting that cell endogenous Wip1 does physiologically reduce p53 function in primary cells (Figures 3D and E). This regulation of p53 by Wip1, however, does not occur at the level of transcription because there was no statistically significant difference in the amounts of p53 mRNA in *Wip1*^*+/+*^*versus Wip1*^*−/−*^ MEFs (Figure 3F).

### Wip1 deficiency reduces Tax-tumorigenesis

**Figure 4:**
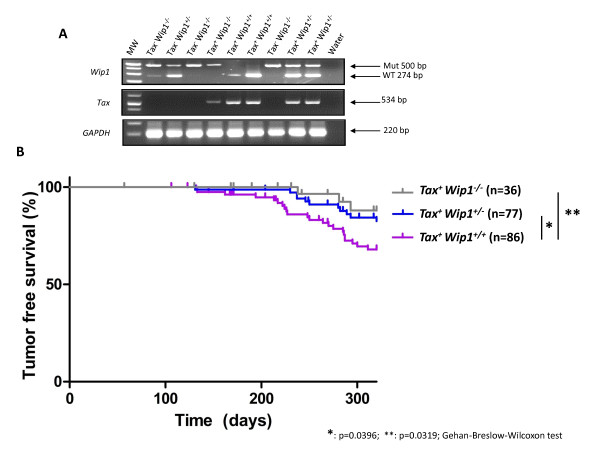
**Statistically significant increase in tumor free survival in *Tax***^***+***^***Wip1***^***−/−***^**mice compared to *Tax***^***+***^***Wip1***^***+/+***^**mice.** (**A**) Genotyping of *Wip1/Tax Tg* mice. The primers for Wip1 distinguish WT and mutant Wip1 alleles with PCR products of 274 and 500 bp, respectively. The GAPDH gene serves as an internal PCR control (220 bp). (**B**) Tumor-free survival curves show increased tumor free survival of *Tax*^*+*^*Wip1*^*−/−*^ and *Tax*^*+*^*Wip1*^*+/−*^ mice compared to *Tax*^*+*^*Wip1*^*+/+*^ animals. Statistically significant (*=p=0.0396 ; **=0.0319) differences between *Tax*^*+*^*Wip1*^*−/−*^ or *Tax*^*+*^*Wip1*^*+/−*^ and *Tax*^*+*^*Wip1*^*+/+*^ mice were determined using Gehan-Breslow-Wilcoxon test.

The above results show that both Wip1 and Tax inactivate p53 function. Next, we asked how the two events might cooperate in tumorigenesis. To address their functional collaboration, we crossed Tax transgenic mice with *Wip1*^*+/+*^ or *Wip1*^*−/−*^ mice. Various genotypic offsprings were obtained from these crosses (genotyping examples are shown in Figure 4A), and the animals were monitored for tumorigenesis over 300 days (Figure 4B). Interestingly, *Wip1*^*+/−*^ and *Wip1*^*−/−*^ mice that express Tax showed significantly better tumor-free survival than *Wip1*^*+/+*^ animals that express Tax (Figure 4B). Indeed, tumor-free survivals were statistically different between *Tax*^*+*^*Wip*^*−/−*^ (p=0.0319; Gehan-Breslow-Wilcoxon test) or *Tax*^*+*^*Wip1*^*+/−*^ mice (p=0.0396; Gehan-Breslow-Wilcoxon test) compared to *Tax*^*+*^*Wip1*^*+/+*^ mice. In view of findings above that p53 activity is higher in *Wip1*^*−/−*^ MEFs compared to *Wip1*^*+/+*^ MEFs; one interpretation of these *in vivo* tumor results is that homozygous loss of Wip1 (i.e.*Tax*^*+*^*Wip1*^*−/−*^) reduces the level of p53-inactivation in Tax expressing cells compared to counterpart cells that expresses both Wip1 and Tax (i.e. *Tax*^*+*^*Wip1*^*+/+*^); this reduced inactivation of p53 could explain the increased tumor-free survival observed in the *Tax*^*+*^*Wip1*^*−/−*^ over the *Tax*^*+*^*Wip1*^*+/+*^ mice.

### Tax expression does not increase Wip1 transcription

**Figure 5:**
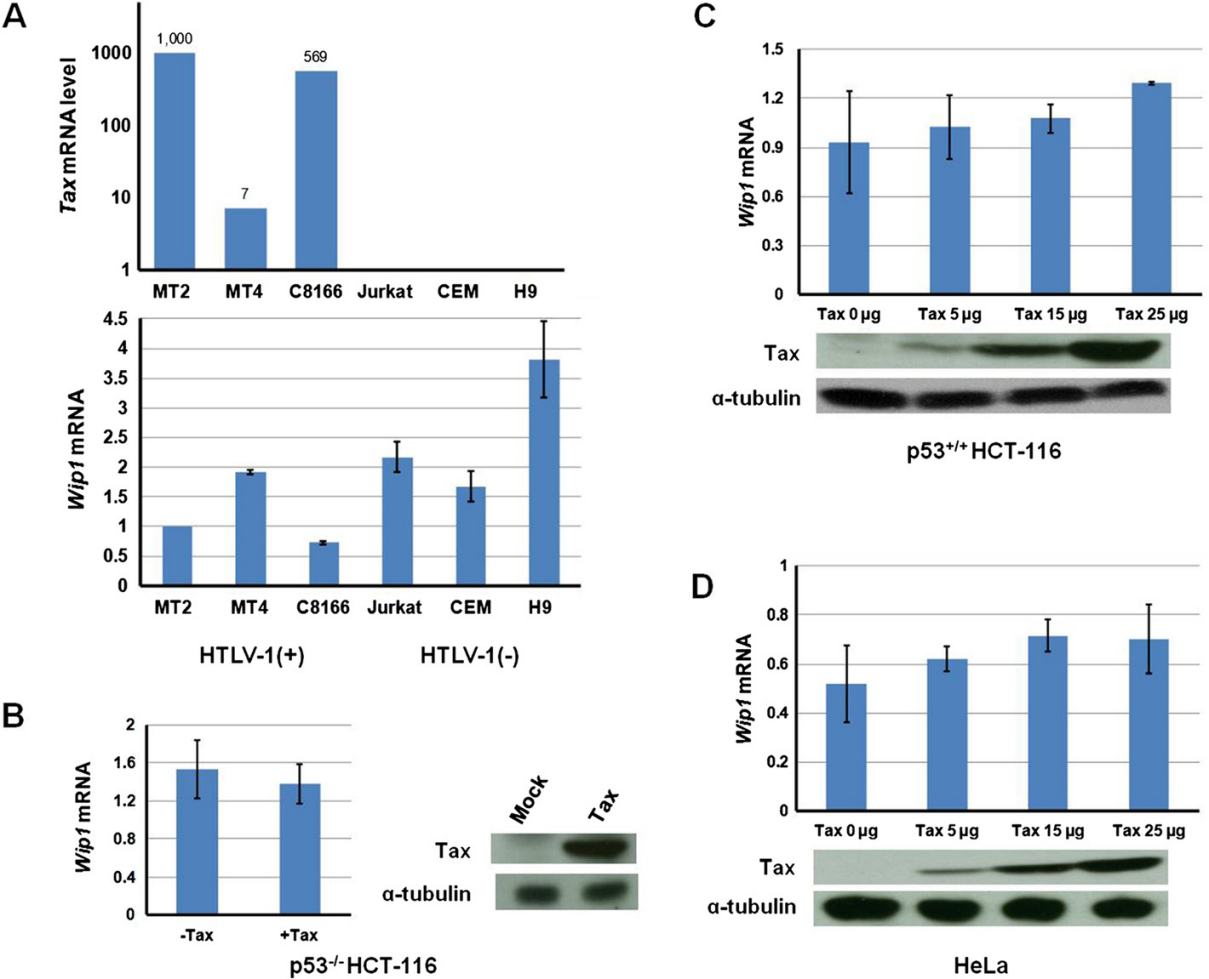
**Analysis of *Wip1 *mRNA expression in Tax-expressing and Tax-non-expressing cells.** (**A**) Total RNAs from HTLV-1–transformed MT-2, MT4, C8166 T-cell lines and HTLV-1-negative CD4^+^ control T-cell lines (Jurkat, CEM, and H9) were extracted and reverse transcribed. The cDNAs were used for real-time RT-PCR analyses of *Wip1*, *Tax*, and *GAPDH* (internal standard) transcripts. The mRNA relative expression levels of *Wip1* and *Tax* mRNA were determined and normalized as multiples of the *GAPDH* mRNA. The columns represent the average results from 3 experiments; the error bars are mean errors. (**B**) Real-time RT-PCR analyses of *Wip1* and *GAPDH* (internal control) transcripts were performed in p53^−/−^ HCT116, (**C**) p53^+/+^ HCT116 and (**D**) HeLa cells after transfection with a control vector or a Tax-expression vector. To detect Tax protein, immunoblots were stained using Tax and α-tubulin specific monoclonal antibodies. Tubulin was used as a loading control.

**Figure 6:**
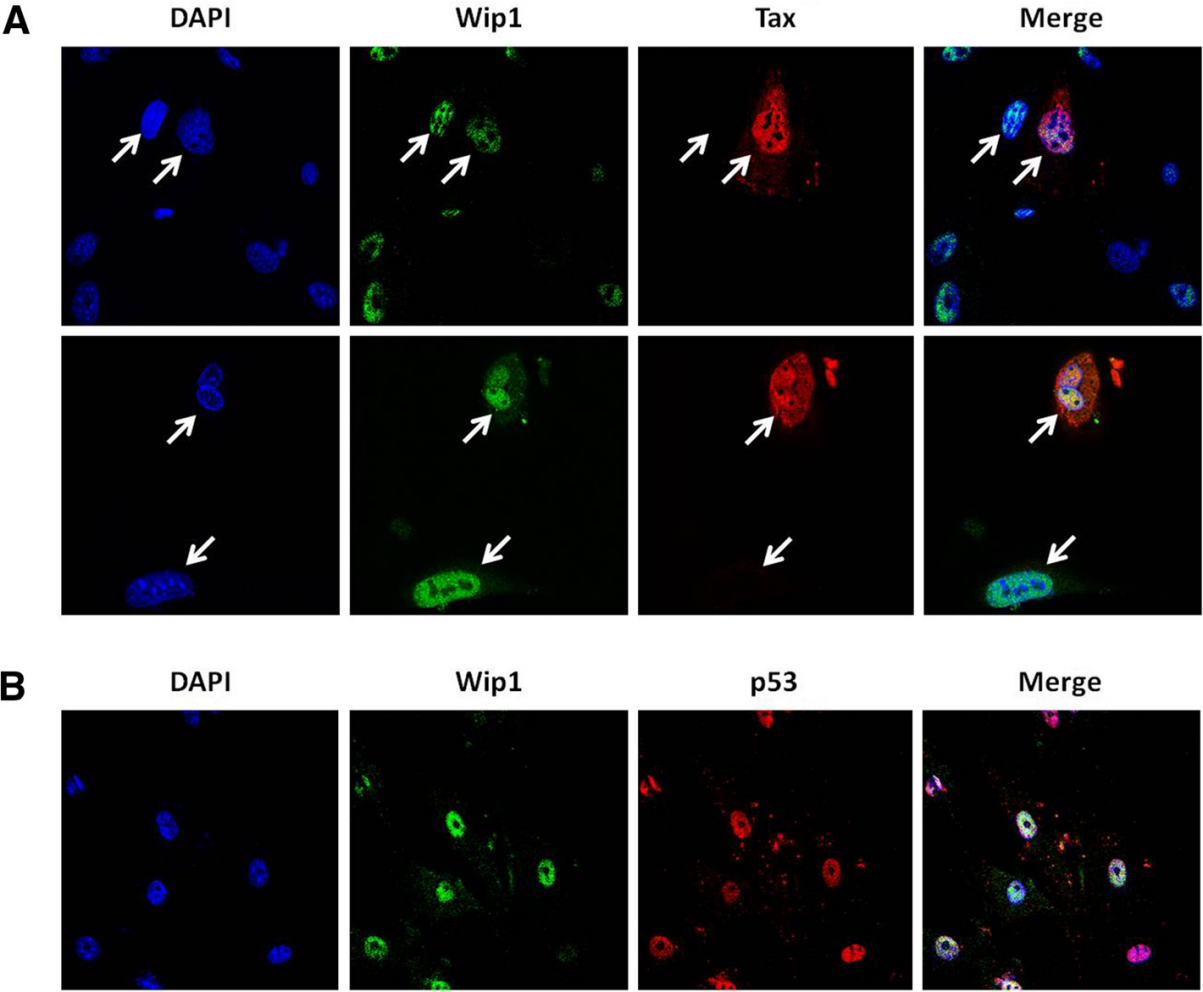
**Confocal analyses of p53, Wip1 and Tax in MEF cells.** (**A**) Analysis of cell endogenous Wip1 and Tax expression and localization by immunofluorescence staining in MEF cells transfected with a Tax expression plasmid for 48 hours. Cells were stained with anti-Tax (red) and anti-Wip1 (green) antibodies. The nuclei were stained with DAPI (blue). Arrows point to cell that expresses Tax (red) and a neighboring cell that does not express Tax. The same two cells are shown to express equal intensities of Wip1 (green). DAPI (blue) stains cellular nuclei. (**B**) The colocalization of cell endogenous p53 and Wip1 in MEF cells. Cells were stained with anti-p53 (red) or anti-Wip1 (green) antibodies, and DAPI was used to stain nuclei (blue).

Figure 4B shows that when Tax and Wip1 are expressed together overall *in vivo* transforming potential is increased. Tax is known to activate or repress the transcription of various genes [75-80]; thus a possibility is that Tax expression affects Wip1 transcription. To address this possibility, RNA was isolated from Tax-expressing HTLV-1–transformed MT2, MT4, C8166 cells and compared to RNAs from HTLV-1–negative CD4^+^ T-cell lines, CEM, Jurkat and H9; specific transcripts were quantified by real-time RT-PCR (Figure 5A). The real-time RT-PCR results showed no correlation between *Tax* expression and *Wip1* expression in these cells. To check in a different way that Tax has no effect on *Wip1* transcription, we transiently transfected p53^−/−^HCT116 (Figure 5B), p53^+/+^HCT116 (Figure 5C), or HeLa cells (Figure 5D) with various amounts of a Tax expression plasmid and measured Wip1 mRNA. p53^−/−^ HCT116 and p53^+/+^ HCT116 cells [81] have been commonly used to study p53 function. In these cells, we observed no statistically significant change in *Wip1* mRNA upon Tax expression. We also transfected MEFs and HCT-116 cells with a Tax expression plasmid and immunostained the cells for Tax and Wip1 proteins. Based on visualization by confocal microscopy, no difference in Wip1 signal intensity was seen in Tax-expressing cells *versus* Tax-negative cells (Figure 6A and Additional file 2: Figure S2A). These findings demonstrate that Tax expression does not change ambient Wip1 protein level and agree with the RNA measurement results that Tax expression does not alter Wip1 mRNA expression (Figure 5).

In our immunostainings, we did note that Tax and Wip1 colocalize in the nucleus (Figure 6A and Additional file 2: Figure S2A). Moreover, additional immunostainings also show that Wip1 and p53 colocalize in the nucleus (Figure 6B and Additional file 2: Figure S2B). Thus, conceivably, Tax, p53, and Wip1 interaction occurs through intranuclear contacts. Currently, we do not have sufficient data to fully understand whether the colocalization of Tax, Wip1, and p53 manifests in direct protein-protein interactions or the proteins interact through bridging by additional factors. Experiments are in progress to define better these mechanistic interactions.

## Discussion

Colloquially known as the guardian of the genome, p53 is an important player in cancer biology, as exemplified by its ubiquitous loss of function in cancers. Thus, approximately 50% of human cancers are genetically mutated in p53 [29,82-85], and the other 50% show attenuated or abrogated p53 activity through means other than mutation [86]. In the case of ATLL, the frequency of p53 gene deletion and mutation is lower than in many other types of cancers and has been reported to approximate 15% [54]. Indeed, our own anecdotal findings are consistent with this low prevalence; in a recent survey of 7 primary ATLL cells, we found no evidence for any of the 11 most frequent p53 somatic gene mutations that have been described for lymphoid neoplasms (Zane, data not shown).

Cancers that retain wild-type p53 gene, nevertheless, can have attenuated p53 activity *via* other mechanisms. For example, Mdm2, an E3 ubiquitin ligase that promotes p53 degradation, is a major negative regulator of p53 [87-89]. Another example of negative regulation arises from the Twist1 protein. Twist1 accumulates in sarcomas that are genotypically p53 wild-type; it dysregulates p53 phosphorylation promoting its degradation [90]. Additional examples come from DNA tumor viruses; some encode proteins that repress p53 activity. Hence, SV40 large T-antigen stabilizes, but inactivates, p53; adenovirus E1B-55-kDa protein, and the E6 oncoprotein of human papilloma virus (HPV) types 16 and 18 target p53 for ubiquitinylation and degradation [91-93]. In the case of HTLV-1, our work here reaffirms previous findings that Tax indeed attenuates p53’s transcriptional activity in cultured cells (Figure 3). However, a perhaps more important implication to arise from our study is that we compare for the first time the impact of Tax inactivation of p53 *versus* p53 inactivation by genetic mutation for their relative contributions to *in vivo* tumorigenesis in mice. To date, it generally has been believed that Tax stringently inactivates p53 activity reducing the need for ATL cells to acquire *p53* inactivating mutations. Our results are, however, incongruent with this notion. Thus, we found that Tax induces tumorigenesis in mice much more robustly in a *p53*^*−/−*^ setting than in a *p53*^*+/+*^ context (Figure 2A), suggesting that Tax inhibition of p53 in the latter context is significantly less complete than p53 inactivation *via* gene mutation. Our findings differ somewhat from those reported by Portis *et al.*[94]. The differences may be due to variances in the mouse numbers, the mouse strains, and the criteria used to determine tumor-free survival and when euthanasias of mice are performed. To date, in the published literature, only cross-sectional findings are associated between p53 genetic mutations and human ATLLs [54]. These findings do not offer clarity on when p53 mutations occurred relative to HTLV-1 infection, Tax expression, and the onset of transformation of ATLL cells. Our results in mice provide prospective analyses of the contribution of a *p53*^*−/−*^ genotype to the initiation of *in vivo* tumorigenesis by Tax. Accordingly, extrapolating our mouse findings to humans suggests that early loss of p53 through a *p53*^*−/−*^ genetic mutation in cells infected by HTLV-1 foretells a worse prognosis compared to a corresponding infection in a counterpart *p53*^*+/+*^ setting.

In our investigation of p53 inactivation, we report for the first time a contributory role by Wip1 in Tax-tumorigenesis. Our insight into the role of Wip1 arose from the observation that loss of Wip1 (i.e. *Wip1*^*−/−*^) significantly reduced the frequency of tumor development in Tax transgenic mice (Figure 4B). We linked this observation to a Wip1-mediated p53 effect because we found that *Wip1*^*−/−*^ MEFs have significantly increased p53 activity over their *Wip1*^*+/+*^ counterparts. Thus, a parsimonious interpretation of the collective findings is that loss of Wip1 phosphatase (i.e. *Wip1*^*−/−*^) increases cell endogenous p53 activity (Figures 3D and E), and this increase in p53 function reduces Tax-tumorigenicity in *Tax*^*+*^*Wip1*^*−/−*^ mice (Figure 4B). Hence, the magnitude of p53 activity is important in regulating the extent of *in vivo* Tax tumorigenesis, and this view is further consistent with the tumor-free survival results comparing *Tax*^*+*^*p53*^*+/+*^ and *Tax*^*+*^*p53*^*−/−*^ mice (Figure 1).

The potential value of inhibiting Wip1 in moderating cancer progression is not only limited to Tax–induced tumors because a Wip1 effect has also been suggested in mammary gland tumors [95], lymphomas [96], colorectal cancers [97], and other spontaneous tumors [98]. Going forward further clarification is needed to understand whether Wip1’s effect on many cancers and its impact on Tax-driven tumor formation are primarily due to its effect on p53 signaling or may also arise from its known effects on other pathways, such as ARF, ATM, and p38 MAPK signaling [96,99]. Studies that compare the *in vivo* tumorigenesis frequencies seen in *Tax*^*+*^*Wip1*^*−/−*^*p53*^*−/−*^ versus *Tax*^*+*^*Wip1*^*+/+*^*p53*^*−/−*^ mice (two genotypes currently being bred in our laboratory) may help to address whether Wip1 has important substrates other than p53 that contribute to Tax-mediated transformation. In other models of carcinogenesis, it has been shown that the singular over-expression of Wip1 is insufficient to initiate oncogenesis [100] and that Wip1 mostly promotes tumors by cooperating with known oncogenes [100]. Nevertheless, amplification of the *Wip1* gene has been described for numerous human primary tumors [101-112], with virtually all such tumors being genetically p53 wild-type [71,72,113]. Based on this observation, one wonders if the low selective pressure for p53 mutations in ATLL could be due to *Wip1* gene amplification in these cells. To our knowledge, this important question has not yet been investigated in ATLLs.

## Conclusions

In summary, despite much progress in HTLV-1 research over the past three decades [114], a salient finding to emerge from this work is the new identification of Wip1 as a cooperating cellular co-factor of Tax in p53-inactivation and *in vivo* tumorigenesis. Currently, our confocal imaging results suggest a colocalization between Tax, Wip1, and p53 within the nucleus (Figure 6 and Additional file 2: Figure S2), but we still lack sufficient data to decipher mechanistically how Tax and Wip1 cooperate to inactivate p53. Amongst several plausible mechanisms, we remain unable to conclude whether Tax can increase Wip1 dephosphorylation of p53 and/or MDM2, a major inhibitor of p53 that has been reported to also be a target of Wip1 [99]. Nonetheless, the functional delineation here of a contribution by Wip1 to Tax tumorigenesis (Figure 4B) does raise the possibility that future uses of small molecule Wip1 phosphatase-inhibitors [115] may benefit ATLL treatment.

## Methods

### Animals and genotyping

The *Tax* and *Wip1*+/− transgenic mice were previously described [15,74]. The *p53*-mutant mice were purchased from the Jackson lab (strain:B6.129S2-*Trp53tm1Tyj*/J) [68]. The Wip1 and p53 knockout and *Tax* transgenic mice were all generated in C57BL/6 × 129/sv backgrounds [15,68,74]. Genotypes of the mice were determined by polymerase chain reactions (PCRs) using primers: *Tax* (Tax-F-7511-7530: 5^′^-tcggctcagctctacagttc-3^′^; Tax-R-8044-8025: 5^′^-tgagggttgagtggaacgga-3^′^), *p53* (wt: 5^′^-acagcgtggtggtaccttat-3^′^, mutant: 5^′^-ctatcaggacatagcgttgg-3^′^ and common: 5^′^-tatactcagagccggcct-3^′^) and *Wip1* (Wip1 Exon4 F: 5^′^-gtggagctatgatttcttcagtgg-3^′^; Wip1 Exon4 R: 5^′^-gatacgacacaagacaaacctcc-3^′^; Wip1 intron 3: 5^′^-acaagcttgcagggctgtttgtgg-3^′^; PGK promoter: 5^′^-cttcccagcctctgagcccagaaagc-3^′^). Experimental research on mice follows NIH approved animal study protocols and guidelines.

### Analyses of pathologies

Mice were necropsied and examined by mouse pathologists. All of the internal organs (spleen, liver, pancreas, kidney, stomach, intestine, lung, heart, brain, lymph node, thyroid gland) were fixed, paraffin embedded, sectioned and stained with H&E for analyses. Tissues that were found to be grossly abnormal at time of necropsy were multiply sectioned and stained by H&E (hematoxylin and eosin) for microscopic histological analyses.

### Cells and reagents

Human cervical cancer cell line HeLa and human colorectal carcinoma cell lines p53^+/+^HCT116 and p53^−/−^HCT116 [81] were cultured in Dulbecco’s modified Eagle’s medium containing 10% fetal bovine serum (FBS) and antibiotics. Human T cell lines MT2, MT4, C8166, Jurkat, A301, CEM, and H9 were maintained in RPMI 1640 with 10% FBS.

### Antibodies

Mouse monoclonal anti-Tax (NIH AIDS Research and Reference Reagent Program) was used to detect Tax protein in immunoblotting and by confocal microscopy. Anti-Flag monoclonal antibody (M2; mouse; Sigma), anti-Wip1 polyclonal antibody (rabbit; Santa Cruz), anti-p53 monoclonal antibody (mouse; Cell Signaling) and anti-tubulin monoclonal antibody (DM1A; mouse; Sigma) were purchased.

### Plasmids and transfections

pG13-Luc, p53 (human wild type) (gifts from B. Vogelstein) and Wip1 (gift from L.A. Donehower) expression plasmids were previously described [73,116,117]. HeLa or p53^+/+^ HCT116 or p53^−/−^HCT116 cells were seeded into twelve-well tissue culture plates for the luciferase assays and into 10 cm-dishes for Tax transfections. Transfections were performed 24 h later, using Lipofectamine and Plus reagent (Invitrogen) as described by the manufacturer. At 24 h after transfection of the reporters, cell lysates were subjected to luciferase assay. Total amounts of DNA to be transfected were adjusted by the addition of empty vectors. To detect luciferase and β-Gal activity, luciferase substrate (Promega) and the Galacto-Star assay system (Applied Biosystems) were used. Relative values of luciferase activity were calculated using β-Gal activity as an internal control for transfection.

### Real-time PCR

For real-time quantitative reverse transcriptase–polymerase chain reaction (qRTPCR), total cellular RNA from samples was isolated using TriZol reagent according to the manufacturer’s instructions (Invitrogen Life technologies). Before reverse transcription, RNA was treated by DNase (Invitrogen) to prevent DNA contamination. First-strand cDNA was synthesized from 1 μg RNA using oligodT and Superscript III reverse transcriptase (Invitrogen). RNA concentration and purity were determined by UV spectrophotometry (nanodrop). The primer pairs were designed using the Universal Probe Library website (Roche diagnostics) (Wip1-L hs: 5^′^-cccatgttctacaccaccagt-3^′^; Wip1-R hs: 5^′^-tggtccttagaattcacccttg-3^′^; p53-L hs: 5^′^-ccccagccaaagaagaaac-3^′^; p53-R hs: 5^′^-aacatctcgaagcgctcac-3^′^; p21-L hs: 5^′^-cgaagtcagttccttgtggag-3^′^; p21-R hs: 5^′^-catgggttctgacggacat-3^′^). The primers of each pair were located in different exons to avoid genomic amplification. Primer and probe sequences to detect Tax in human T-cells [118] and Tax-SK43: 5^′^-cggatacccagtctacgtgt-3^′^ and Tax-SK44: 5^′^-gagccgataacgcgtccatcg-3^′^ to detect Tax in mouse spleens. GAPDH was used as the reference gene for he normalization of results (GAPDH-R: 5^′^-agtgggtgtcgctgttgaag-3^′^; GAPDH-F: 5^′^- tggtatcgtggaaggactca-3^′^). PCRs were performed using iQSupermix (Bio-Rad) (for quantification of Tax cDNAs in human T-cells) and iQSYBR Green Supermix (for quantification of other cDNAs) on a CFX96 system (Bio-Rad). A large amount of cDNA was prepared from the MT2, C8166, and MT4 cell lines prior to the experiment. This cDNA was 10 fold-diluted, aliquoted and used as a calibrator for Tax and other RT-PCR runs, respectively. For relative quantification and normalization, the comparative Ct (or Eff-DDC) method was used [119].

### Immunofluorescence

Cells were cultured on glass coverslips, and fixed in 4% paraformaldehyde at 24 h after transfection. After blocking of nonspecific reactions with 1% bovine serum albumin (BSA), cells were then incubated with the indicated primary antibodies, followed by a subsequent incubation with the secondary antibodies conjugated with Alexa Fluor 488 or 594 (Molecular Probes). DNA was counterstained with 0.1 μg/ml Hoechst 33342. Coverslips were mounted in Prolong Antifade (Molecular Probes), and cells were visualized with a Leica TCS SP2 confocal microscope.

### Statistical analyses

The statistical analysis of tumor numbers, survival curves, and spleen weights were computed using the PRISM software (version 5.03).

## Competing interests

The authors declare that they have no competing interests.

## Authors' contributions

LZ designed and performed the work, analyzed the data and wrote the paper. YJ started mouse breedings and genotypings. YM performed some genotypings. VY, SWT and CYC contributed reagents and technical advice for the work and edited the paper. LR and XL provided, respectively, Tax and Wip1 mice and participated in discussions. KTJ conceived of the study and supervised the work and wrote the paper. All authors read and approved the final manuscript.

## Supplementary Material

Additional file 1 Figure S1 Analyses of *Tax* mRNA expression in Tax^+^ p53^−/−^ and Tax^+^ p53^+/+^ mouse spleen tissues. Total RNAs from mouse spleen tissues were extracted and reverse transcribed. The cDNAs were used for real-time RT-PCR analyses of Tax and GAPDH (internal standard) transcripts. The mRNA relative expression levels of Tax mRNA were determined and normalized as multiples of the GAPDH mRNA. There was no statistically significant difference in Tax mRNA expression levels between Tax^+^ p53^−/−^ and Tax^+^ p53^+/+^ mice (p=0.2758; unpaired t-test). Each circle or square represents an independent mouse spleen tissue. Click here for file

Additional file 2 Figure S2 Confocal analyses of p53, Wip1 and Tax in MEF cells. (A) Analysis of cell endogenous Wip1 and Tax expression and localization by immunofluorescence staining in HCT-116 cells transfected with a Tax expression plasmid for 48 hours. Cells were stained with anti-Tax (red) and anti-Wip1 (green) antibodies. The nuclei were stained with DAPI (blue). Arrows point to cell that expresses Tax (red) and a neighboring cell that does not express Tax. The same two cells are shown to express equal intensities of Wip1 (green). DAPI (blue) stains cellular nuclei. **(B)** The colocalization of cell endogenous p53 and Wip1 in HCT-116 cells. Cells were stained with anti-p53 (red) or anti-Wip1 (green) antibodies, and DAPI was used to stain the nuclei (blue). (JPEG 142 kb) Click here for file
