# Supplementary figures and images for: Wip1 and p53 contribute to HTLV-1 Tax-induced tumorigenesis
Source: Retrovirology. 2012 Dec 21;9:114. doi: 10.1186/1742-4690-9-114 (PMC3532233; doi:10.1186/1742-4690-9-114)

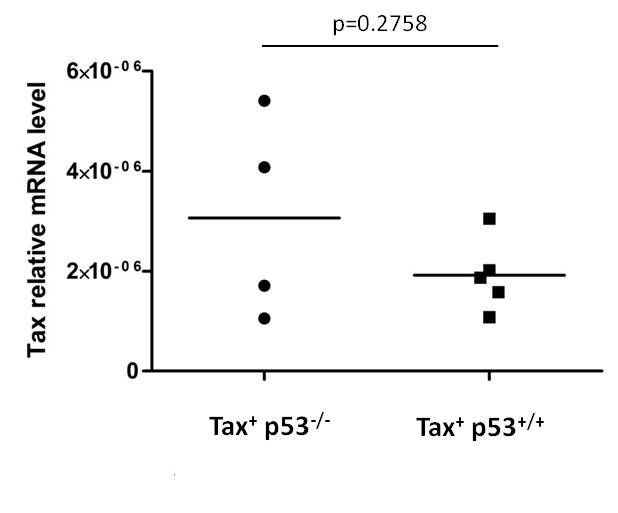

Supplement: Additional file 1 Figure S1 — Analyses of Tax mRNA expression in Tax+ p53−/− and Tax+ p53+/+ mouse spleen tissues. Total RNAs from mouse spleen tissues were extracted and reverse transcribed. The cDNAs were used for real-time RT-PCR analyses of Tax and GAPDH (internal standard) transcripts. The mRNA relative expression levels of Tax mRNA were determined and normalized as multiples of the GAPDH mRNA. There was no statistically significant difference in Tax mRNA expression levels between Tax+ p53−/− and Tax+ p53+/+ mice (p=0.2758; unpaired t-test). Each circle or square represents an independent mouse spleen tissue. [file 1742-4690-9-114-S1.jpeg]

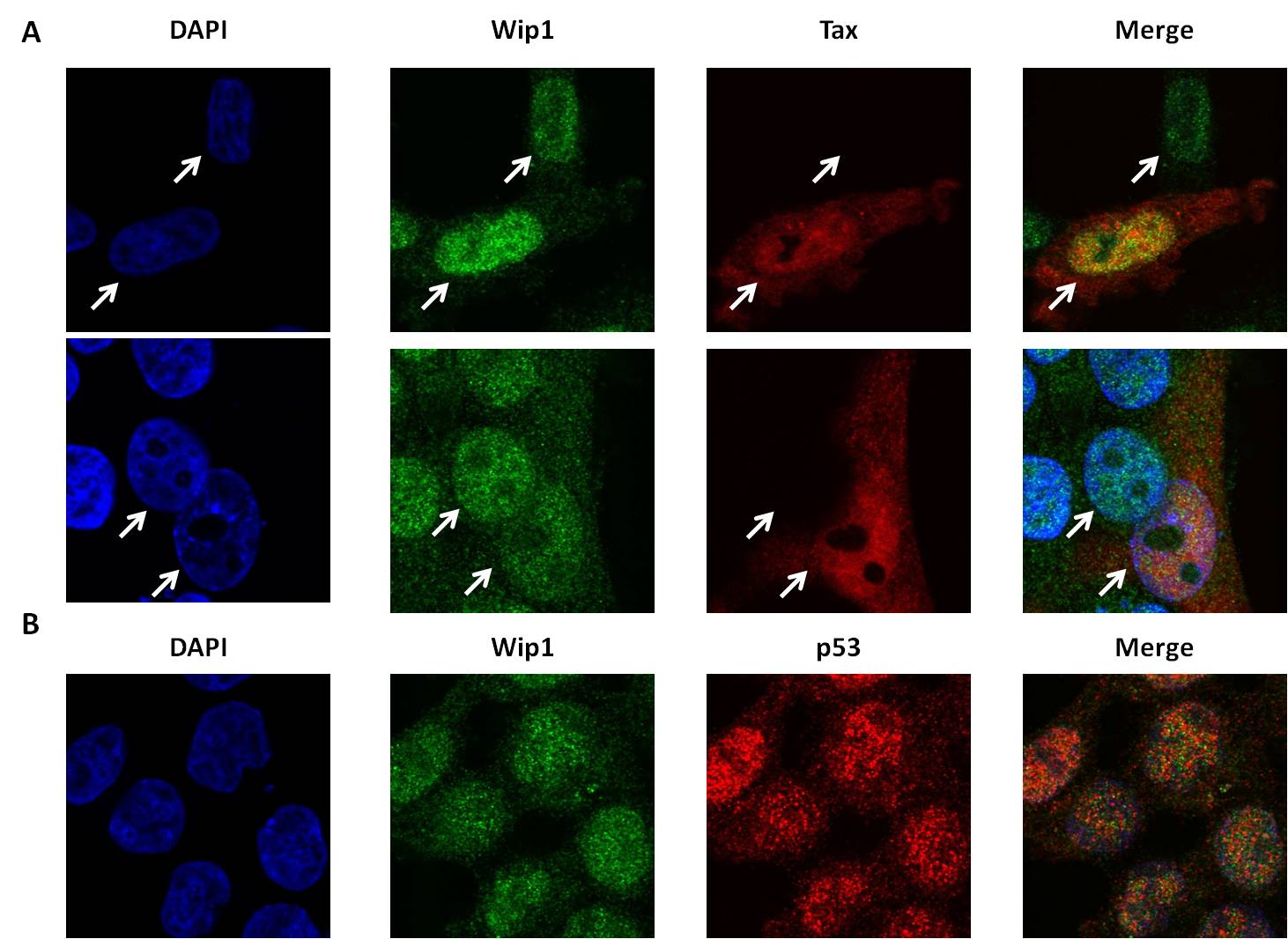

Supplement: Additional file 2 Figure S2 — Confocal analyses of p53, Wip1 and Tax in MEF cells. (A) Analysis of cell endogenous Wip1 and Tax expression and localization by immunofluorescence staining in HCT-116 cells transfected with a Tax expression plasmid for 48 hours. Cells were stained with anti-Tax (red) and anti-Wip1 (green) antibodies. The nuclei were stained with DAPI (blue). Arrows point to cell that expresses Tax (red) and a neighboring cell that does not express Tax. The same two cells are shown to express equal intensities of Wip1 (green). DAPI (blue) stains cellular nuclei. (B) The colocalization of cell endogenous p53 and Wip1 in HCT-116 cells. Cells were stained with anti-p53 (red) or anti-Wip1 (green) antibodies, and DAPI was used to stain the nuclei (blue). (JPEG 142 kb) [file 1742-4690-9-114-S2.jpeg]
